# Supplementary material for: EnzML: multi-label prediction of enzyme classes using InterPro signatures
Source: BMC Bioinformatics. 2012 Apr 25;13:61. doi: 10.1186/1471-2105-13-61 (PMC3483700; doi:10.1186/1471-2105-13-61)
Supplement: Addtional file 5 — The Java code to format the data files, evaluate and predict. The file enzml_java_code.tar.gz contains the Java code used to format database data to ARFF and XML formats, to execute cross and train-test (jackknife) evaluations and to record evaluation results to database. More information is included in the readme.txt file and the Javadoc files. The code can be used with a MySQL database. To use a different database software, other JDBC drivers might be required. [file 1471-2105-13-61-S5.gz › java_code/enzml2011/doc/uk/ac/ed/inf/csb/taweka/test/mulan/MulanExperimentDbWriter.html]

MulanExperimentDbWriter


---


|  |  |  |
| --- | --- | --- |
| **Overview** \| **Package** \| **Class** \| **Use** \| **Tree** \| **Deprecated** \| **Index** \| **Help** | |  |
| **PREV CLASS**   **NEXT CLASS** | **FRAMES**    **NO FRAMES**     **All Classes** |  |
| SUMMARY: NESTED \| FIELD \| CONSTR \| METHOD | DETAIL: FIELD \| CONSTR \| METHOD |  |


---


## uk.ac.ed.inf.csb.taweka.test.mulan Class MulanExperimentDbWriter

```
java.lang.Object
  uk.ac.ed.inf.csb.taweka.test.mulan.MulanExperimentDbWriter
```

---

``` public class MulanExperimentDbWriter extends java.lang.Object ```

Class

**Version:**
:   8 Jun 2010

**Author:**
:   Luna De Ferrari luna.deferrari-at-ed.ac.uk

---

**Constructor Summary**

`MulanExperimentDbWriter(uk.ac.ed.inf.csb.utils.databaseutils.DbManager dbManager,
MulanEvaluator experiment)`
  
          Constructor 1: initialises the table (uses given database connection
file)


| **Method Summary** | |
| --- | --- |
| `void` | `createTable()`             Creates the table for the ARFF file records |
| `void` | `dropAndCreateTable()`             Beware! This method drops and recreates the ARFF table. |
| `ArffGenerator` | `getArffGenerator()` |
| `int` | `getArffRecordId()` |
| `java.lang.String` | `getPropertyFromTable(java.lang.String propertyName)` |
| `uk.ac.ed.inf.csb.utils.databaseutils.Table` | `getTable()` |
| `static void` | `main(java.lang.String[] args)`             Main for recreating table |
| `void` | `savePropertiesToDb()`             Insert the properties in the table, each property value is inserted in the table-field with the same name as the property key |
| `boolean` | `writeTableToDb()` |

**Methods inherited from class java.lang.Object**

`equals, getClass, hashCode, notify, notifyAll, toString, wait, wait, wait`

**Constructor Detail**

### MulanExperimentDbWriter

```
public MulanExperimentDbWriter(uk.ac.ed.inf.csb.utils.databaseutils.DbManager dbManager,
                               MulanEvaluator experiment)
```

:   Constructor 1: initialises the table (uses given database connection
    file)

    **Throws:**: `java.sql.SQLException`


**Method Detail**

### createTable

```
public void createTable()
```

:   Creates the table for the ARFF file records

---


### writeTableToDb

```
public boolean writeTableToDb()
```

---


### savePropertiesToDb

```
public void savePropertiesToDb()
```

:   Insert the properties in the table, each property value is inserted in
    the table-field with the same name as the property key

---


### dropAndCreateTable

```
public void dropAndCreateTable()
```

:   Beware! This method drops and recreates the ARFF table. All arff files
    definitions already in the table will be lost!

---


### getPropertyFromTable

```
public java.lang.String getPropertyFromTable(java.lang.String propertyName)
```

---


### getTable

```
public uk.ac.ed.inf.csb.utils.databaseutils.Table getTable()
```

---


### getArffGenerator

```
public ArffGenerator getArffGenerator()
```

---


### getArffRecordId

```
public int getArffRecordId()
```

---


### main

```
public static void main(java.lang.String[] args)
                 throws java.sql.SQLException
```

:   Main for recreating table

    :   **Parameters:**: `args` - **Throws:**: `java.sql.SQLException`


---


|  |  |  |
| --- | --- | --- |
| **Overview** \| **Package** \| **Class** \| **Use** \| **Tree** \| **Deprecated** \| **Index** \| **Help** | |  |
| **PREV CLASS**   **NEXT CLASS** | **FRAMES**    **NO FRAMES**     **All Classes** |  |
| SUMMARY: NESTED \| FIELD \| CONSTR \| METHOD | DETAIL: FIELD \| CONSTR \| METHOD |  |


---
